# Ultrasound-Guided Continuous Bilateral Erector Spinae Plane Blocks Are Associated with Reduced Opioid Consumption and Length of Stay for Open Cardiac Surgery: A Retrospective Cohort Study

**DOI:** 10.3390/jcm10215022

**Published:** 2021-10-28

**Authors:** Brian N. Vaughan, Cheryl L. Bartone, Catherine M. McCarthy, Geoffrey A. Answini, William E. Hurford

**Affiliations:** 1 Department of Anesthesiology, University of Cincinnati, Cincinnati, OH 45267, USA; william.hurford@uc.edu; 2 The Christ Hospital Health Network, Heart and Vascular Services, Cincinnati, OH 45219, USA; cheryl.bartone@thechristhospital.com (C.L.B.); catherine.mccarthy@thechristhospital.com (C.M.M.); geoffrey.answini@thechristhospital.com (G.A.A.)

**Keywords:** erector spinae plane block, cardiac surgery, regional anesthesia, acute pain, fascial plane block, morphine consumption

## Abstract

This study tested the hypothesis that continuous bilateral erector spinae plane blocks placed preoperatively would reduce opioid consumption and improve outcomes compared with standard practice in open cardiac surgery patients. Patients who received bilateral continuous erector spinae plane blocks for primary open coronary bypass, aortic valve, or ascending aortic surgery were compared to a historical control group. Patients in the block group received a 0.5% ropivacaine bolus preoperatively followed by a 0.2% ropivacaine infusion begun postoperatively. No other changes were made to the perioperative care protocol. The primary outcome was opioid consumption. Secondary outcomes were time to extubation and length of stay. Twenty-eight patients received continuous erector spinae plane blocks and fifty patients served as historic controls. Patients who received blocks consumed less opioids, expressed as oral morphine equivalents, both intraoperatively (34 ± 17 vs. 224 ± 125 mg) and during their hospitalization (224 ± 108 vs. 461 ± 185 mg). Patients who received blocks had shorter times to extubation (126 ± 87 vs. 257 ± 188 min) and lengths of stay in the intensive care unit (35 ± 17 vs. 58 ± 42 h) and hospital (5.6 ± 1.6 vs. 7.7 ± 4.6 days). Continuous erector spinae plane blocks placed prior to open cardiac surgical procedures reduced opioid consumption, time to extubation, and length of stay compared to a standard perioperative pathway.

## 1. Introduction

Open cardiac procedures historically relied upon large doses of opioids to provide hemodynamic stability during surgery and analgesia in the postoperative period [1]. While opioids are useful for their analgesic effect, their use is associated with adverse events that can not only harm patients but also increase the cost of care and length of stay (LOS) [2]. In addition, opioid analgesia puts patients at risk for developing long-term persistent use after discharge, further increasing morbidity from opioid therapy [3]. On average, it has been reported that 3% of patients undergoing major elective surgery will become persistent users of opioids [3]. The risk for persistent use after cardiac surgery is even higher, averaging 8% in patients following coronary artery bypass grafting [4]. Importantly, as the risks of opioid therapy are dose related [2,4], reducing opioid consumption in the perioperative period should, in theory, improve patient outcomes [5,6]. Reducing opioid consumption, however, must be accomplished without compromising analgesia since higher levels of pain in the postoperative period place patients at risk for developing chronic pain syndromes [7].

Although regional anesthesia/analgesia reduces opioid consumption in the perioperative period [6], regional techniques have not been widely adopted for cardiac surgery due to the perceived risk of epidural and paravertebral block placement in the face of the anticoagulation necessary for cardiopulmonary bypass [8]. Seeking to find a more superficial and, thus, theoretically safer approach to providing analgesia to the thoracic wall, Forero and coworkers proposed the erector spinae plane (ESP) block [9]. This description was followed by numerous case reports and small case series suggesting ESP blocks could provide analgesia for many indications, such as rib fractures [10], ventral hernias [11], and breast surgery [12]. Recent retrospective and prospective studies have added to the level of evidence supporting the efficacy of ESP blocks [13].

Initial studies examining ESP blocks for cardiac surgery patients have shown promising results. Patients who received single-injection ESP blocks preoperatively had shorter durations of mechanical ventilation and had shorter intensive care unit (ICU) lengths of stay compared to patients receiving intravenous paracetamol and tramadol [14]. Patients receiving single-injection ESP blocks also had improved pain scores in the immediate postoperative period, although the analgesic benefits began to fade within eight hours of extubation [14]. Nagaraja and colleagues, comparing continuous epidural analgesia with continuous ESP blocks, reported patients having a similar need for rescue analgesia and similar times to extubation and intensive care unit lengths of stay [15]. When comparing continuous ESP blocks to an institution’s fast track protocol, patients who received blocks consumed less opioids, had a shorter time to drain removal and first mobilization, and had lower pain scores at rest at one month [16]. Accordingly, we hypothesized that bilateral continuous ESP blocks placed prior to open cardiac surgery will decrease perioperative opioid consumption and improve patient outcomes as measured by time to extubation and LOS. We designed a retrospective cohort study to examine the effect of continuous ESP blocks placed preoperatively on patients having open cardiac surgery compared to matched historical controls treated with standard practice without regional analgesia.

## 2. Materials and Methods

This retrospective cohort study was approved by The Christ Hospital Institutional Review Board (IRB #19–85) on 27 December 2019. Since the study posed no more than minimal risk, waiver of informed consent was granted.

### 2.1. Subject Selection

The retrospective intervention group was composed of patients who received preoperative continuous ESP blocks for open cardiac surgery by two surgeons between 21 June 2019, and 30 October 2019. Patients were included in the study if they were undergoing either elective primary CABG, aortic valve, or ascending aortic surgery and had no history of either chronic lung disease, opioid use, or substance abuse. A matched retrospective control group was composed of patients undergoing similar elective open cardiac surgical procedures by the same surgeons in the two years prior to the institution of continuous ESP blocks (June 2019). Controls were matched with the intervention group by surgeon, type of surgery, age, sex, smoking status, and cardiac function as measured by left ventricular ejection fraction and excluded by the presence of pre-existing lung disease, prior opioid use, or substance abuse. Researchers were blinded to intraoperative and postoperative data during the matching of controls.

### 2.2. Data Collection

Sociodemographic data collected from electronic medical records included: age, sex, body mass index, smoking status, and cardiac function as defined by left ventricular ejection fraction. Surgical data collected included: procedure time, cardiopulmonary bypass time, and cross-clamp time. Outcome data collected included: opioid consumption; non-opioid analgesic consumption; time to extubation, ICU LOS, and hospital LOS; and the patient’s highest pain score reported on each postoperative day. All data were collected from The Christ Hospital electronic medical record, and there were no missing data for any participants. Opioids consumed were converted to oral morphine equivalents (OME) using the National Drug and Alcohol Research Center and Centers for Disease Control guidelines [17,18].

### 2.3. Technique for Erector Spinae Plane Blocks

**Figure 1:**
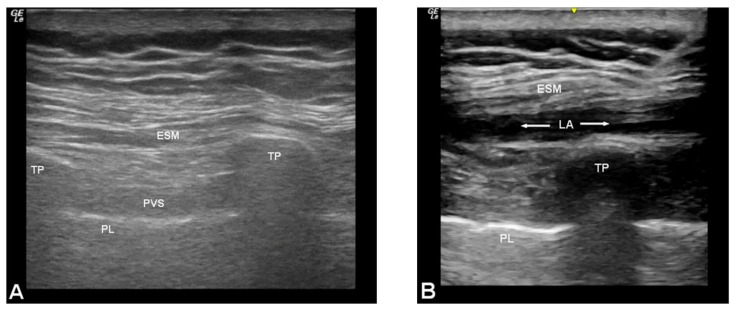
(**A**) Ultrasound anatomy of the erector spinae plane (ESP) prior to block placement. (**B**) Ultrasound demonstrating local anesthetic (LA) spread in the ESP. Abbreviations: ESM, erector spinae muscle; PL, pleura; PVS, paravertebral space; TP, transverse process.

Patients in the intervention group received bilateral continuous ESP blocks placed preoperatively by one of three anesthesiologists with experience placing ESP catheters. With the patient in the sitting position, the skin was prepped bilaterally with chlorhexidine, and a sterile field was created. Using ultrasound (GE LOGIQ *e* with a 12L-RS 4.2-13 MHz linear transducer; GE Healthcare, Chicago, IL, USA), the T4 and T5 transverse processes and overlying erector spinae muscle and intertransverse ligament were identified (Figure 1A).

Under continuous ultrasound guidance, a 17 G, 3.5 inch Tuohy (Arrow, Morrisville, NC) was advanced in a caudal-to-cranial direction towards the apex of the T5 transverse process. Normal saline was injected via the needle to confirm tip placement in the plane between the fascia of the erector spinae muscle and the intertransverse ligament. Once correct placement was confirmed, a bolus of 25 mL 0.5% ropivacaine was injected (Figure 1B), and a 19 G nerve block catheter (Arrow) was placed 3–5 cm past the tip of the needle in the space created by the hydrodissection. After catheter placement, 5 mL 0.5% ropivacaine was injected via the catheter to confirm functionality. The process was repeated for the contralateral side. Intraoperatively, opioid analgesics were dosed by the anesthesia care team by the usual criteria of perceived need, such as pain or anxiety during catheter placement and changes in heart rate, blood pressure, or other factors during the anesthetic. Upon arrival to the intensive care unit, an infusion of 0.2% ropivacaine was begun at 3–7 mL/hour/side and continued until after chest tube removal. Based on patient comfort, the infusion was titrated up to maximum of 7 mL/hour/side and continued for a maximum of six days. Postoperatively, the standard analgesic regimen was followed, and as-needed analgesics were given based on reported pain score and patient request. Other than continuous ESP block placement, no changes were made to the perioperative care protocol used at the institution.

### 2.4. Outcomes and Statistical Analysis

Our primary outcome was opioid consumption (OME) over the patient’s hospital course up to and including postoperative day five (i.e., day of surgery plus five days). Our secondary outcomes were intraoperative and postoperative opioid consumption and time to extubation, ICU LOS, and hospital LOS. We performed a power analysis that suggested 80 patients with a 2:1 allocation of control and case groups, respectively, would be required to provide 90% power to detect an 18% difference in our primary outcome variable. In order to increase the ability to detect a difference between the groups, a larger control group was constructed compared to recent studies on ESP blocks for cardiac surgery patients [15,16]. We compared the demographics and clinical characteristics of the two groups. Continuous variables were analyzed using Student’s t-test and Wilcoxon rank sum to account for a lack of normal distribution in several variables (i.e., intraoperative OME, ICU LOS, ventilation time, hospital LOS, and pain scores). We report summative data as means and standard deviations, or median and interquartile ranges as appropriate, for the data distribution. Categorical variables were analyzed using chi-square. Statistical significance was considered to be a *p* value of <0.05. All statistics were performed using R statistics package version 3.6.3 [19].

## 3. Results

A convenience sample of 31 patients receiving continuous ESP blocks over a 4 month period was initially included in the study. Of those 31 patients, three were excluded from statistical analysis: one patient had a revision of an aortic valve replacement; one patient had a combined aortic valve and mitral valve procedure; and one patient was found to have a history of substance abuse. Fifty matched patients served as historic controls. Baseline demographic data and procedure times (Table 1) were similar between the block and control groups.

With the exception of gabapentin, patient consumption of non-opioid analgesics (Table 2) was also similar between the groups. Patients in the control group consumed significantly more gabapentin (*p* = 0.027) than those in the block group with a mean difference of 333 mg.

**Figure 2:**
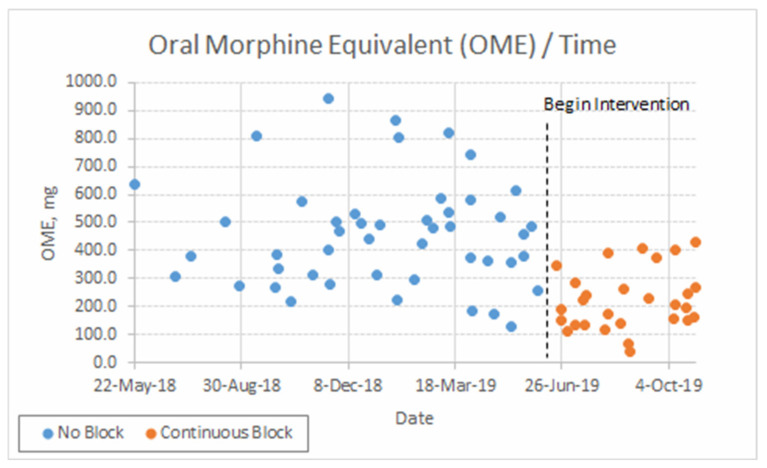
Total opioid consumption during hospitalization through postoperative day 5 per case in mg oral morphine equivalents (OME) over time.

**Figure 3:**
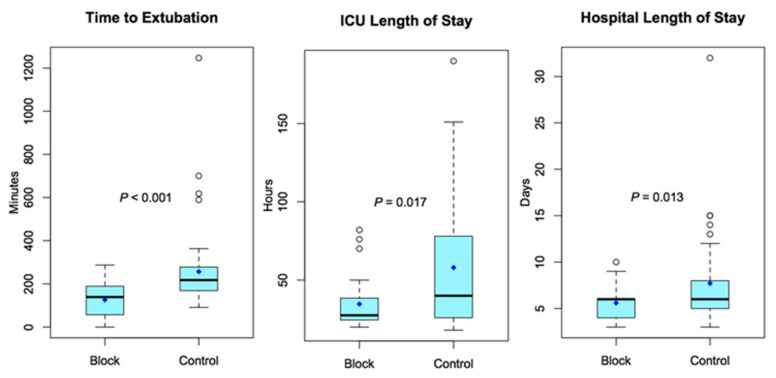
Box and whisker plots represent data as median values (bold horizontal line), interquartile range (box), interquartile range × 1.5 or furthest datum (dotted lines), and any outliers (open circles). Mean values are represented by blue diamonds.

Patients who received blocks consumed less opioids over their hospitalization (Table 2, *p* < 0.001), as expressed in mg oral morphine equivalents. Total opioid consumption for each case over time is illustrated in Figure 2. For secondary outcomes, see Table 2 and Figure 3.

While the block group consumed less opioids intraoperatively (*p* < 0.001), the level of postoperative opioid consumption did not differ significantly (mean difference 48 mg OME, *p* = 0.104). For other secondary outcomes, patients who received blocks had shorter times to extubation (126 ± 87 vs. 257 ± 188 min, *p* < 0.001); intensive care unit LOS (35 ± 17 vs. 58 ± 42 h, *p* = 0.017); and hospital LOS (5.6 ± 1.6 vs. 7.7 ± 4.6 days, *p* = 0.013). There was no difference between the groups in terms of the median worst pain score reported by patients on each day for postoperative days 0–5 (Table 2).

## 4. Discussion

Our study confirms that ESP blocks placed preoperatively for cardiac surgery patients significantly reduce perioperative opioid consumption. In addition, patients who received continuous ESP blocks were extubated approximately 50% faster than those patients who did not receive a block. Intensive care unit LOS was reduced by nearly 1 day and hospital LOS was over 2 days lower compared to those not receiving a block. This study adds to the body of data supporting the use of ESP blocks for cardiac surgery patients. In addition, it reports a decrease not only in ICU length of stay but also in overall hospital length of stay for those patients receiving continuous ESP blocks prior to open cardiac surgery. Similar to previous studies [14,15,16], ESP blocks appear to be a safe technique for postoperative analgesia. There were no block-related complications, such as infection, local anesthetic toxicity, pneumothorax, or hematoma. Clearly, if these results are confirmed to be reproducible and generalizable to all open cardiac procedures, ESP blocks would represent a significant step forward in the care of these patients.

Median sternotomy for cardiac surgery causes moderate-to-severe postoperative pain that has traditionally been treated with high dose opioids [1]. However, reliance on opioids not only leads to adverse events [2] but is also associated with worse patient outcomes, such as prolonged intubation, longer ICU LOS, and longer hospitalizations after cardiac surgery [8], as well as dependence on opioids after discharge [3,4]. The overarching goal of all expedited recovery protocols is to improve outcomes by reducing the negative consequences provoked by the stress of surgery [20]. Cornerstones of expedited recovery protocols include improved pain control and opioid reduction, accomplished via a combination of regional and multimodal analgesia. Expedited recovery protocols in cardiac surgery have been shown to reduce opioid use, reduce ventilator time, and decrease both ICU and overall hospital length of stay [20,21]. Even though improvements have been made in expedited recovery protocols for cardiac surgery, progress has been slower compared to other surgical specialties [21]. Regional techniques have not been widely adopted due to the perceived risk of epidural and paravertebral block placement in the face of the anticoagulation necessary for cardiopulmonary bypass [8,22]. Given the superficial nature of ESP blocks, it has been hypothesized that they would be both a safe and effective analgesic technique for cardiac surgery patients [8,22]. Initial studies demonstrated that the use of ESP blocks can reduce perioperative opioid consumption in cardiac surgery patients [14,15,16]. Since the risk of persistent opioid use rises with increased perioperative opioid consumption [4], reducing perioperative opioid consumption is essential. In addition to reducing opioid consumption, ESP blocks have been reported to improve outcomes, such as a shorter time to extubation, a decreased LOS, and improved pain scores after discharge in cardiac surgery patients [14,15,16]. This study examined the benefit of adding regional analgesia via ESP blocks to expedited recovery within a patient care protocol that already included multimodal analgesics, such as acetaminophen and gabapentin.

Do continuous ESP blocks provide patients with additional benefits compared to single-injection blocks? A direct comparison of continuous to single-injection ESP blocks for cardiac surgery has not been made, although multiple studies have suggested a benefit in other types of surgery [23,24,25]. In our current study, opioid consumption was reduced intraoperatively and over the entire hospitalization. Opioid consumption in the postoperative period was reduced by about 20% in those who received a block, but this decrease was not statistically significant. The confounding influence of the additional gabapentin administered to the control group, the opioid-sparing effect of sedatives due to longer mechanical ventilation in the control group, and the likely carry-over effect into the postoperative period of the significantly larger opioid dose in the control group limit the study’s ability to define the opioid difference in the postoperative period. Based on a recent meta-analysis [26], the mean dose of intravenous (IV) morphine equivalent consumed by those given gabapentinoids was 25.3 mg compared with 38.7 mg in those that did not receive them, equivalent to a difference of 40.2 mg OME. It is likely that the significantly higher use of gabapentinoids reduced postoperative opioid consumption in the control group. In addition, if only 13.7 mg OME, equivalent to less than 3 mL of fentanyl, was, in reality, providing analgesia in the immediate postoperative period, the opioid consumption totals should be adjusted in both time periods. If the intraoperative total was reduced by 13.7 mg OME and added to the postoperative period, the intraoperative difference remains highly significant (*p* < 0.001), while the postoperative difference becomes significant (*p* = 0.04). We would suppose that the combination of these three factors reduced the measured opioid consumption in the postoperative period enough to prevent the detection of a difference between the block and control group. While this study did not investigate the effect of continuous ESP blocks on the development of chronic postoperative pain, Macaire and colleagues reported that those who received continuous ESP blocks had less pain one month after surgery [16]. Ultimately, the quantification of the additional benefits of continuous ESP blocks will need to be answered by a direct comparison with single-injection blocks in a prospective, randomized trial.

There are several limitations to our study. Foremost, the study design was retrospective and not randomized. Given that the block patients were matched with historical controls, selection bias must be considered. As with any quality improvement process, the desire to see positive change can subtly affect other parts of the care process, leading to improvement that is separate from the variable being measured. This is especially true given that the staff were not blinded to the patients that had received blocks. Stated in another way, the fact that hospital staff were aware of the study (i.e., Hawthorne effect) may have altered their behavior and could be a source of confounding. Moreover, natural improvement over time may be a source of the positive outcome seen; however, given the time course of opioid consumption of the control and block groups over time, this is less likely to be the case. As seen in Figure 2, there is a clear change in perioperative opioid consumption that occurred when ESP blocks were instituted. Both the perioperative opioid consumption and the variability among patients were reduced compared with controls. While the blocks were placed by experienced personnel, the lack of objective testing of the block after placement did not permit quantification of the success rate of the block. The inclusion of failed blocks would have increased the amount of opioid consumption in the block group and reduced our effect size. The lack of pain scores, patient satisfaction, and residual pain after discharge also limit our study. Including these variables in future studies would better define the utility and cost benefits of ESP blocks.

## 5. Conclusions

In this study, compared to a standard perioperative pathway, continuous ESP blocks placed prior to open cardiac surgical procedures:

1. Reduced opioid consumption by 238 mg oral morphine equivalents (52%);
2. Reduced time to extubation by 130 min;
3. Reduced intensive care unit LOS by 23.3 h;
4. Reduced overall hospital LOS by 2.1 days.

Continuous ESP blocks appear to be an effective option to decrease opioid consumption and improve outcomes in patients undergoing open cardiac surgery.

## Figures and Tables

**Table 1:** Demographic data and procedural times.

	Control (*n* = 50)	Case (*n* = 28)	Mean Difference	*p*-Value
	Mean ± SD	Mean ± SD	(95% CI)	
Age	65.8 ± 7.7	65.6 ± 8.5	0.2 (−3.7 to 4.1)	0.921
Male (%)	38 (76%)	21 (75%)	-	1.000
BMI	31.1 ± 5.3	29.9 ± 4.7	1.2 (−1.2 to 3.5)	0.326
Smoking status				
Never smoker (%)	23 (46%)	13 (46%)	-	1.000
Former smoker (%)	23 (46%)	12 (43%)	-	0.976
Current smoker (%)	4 (8%)	3 (11%)	-	1.000
Left ventricular ejection fraction	52 ± 8	54 ± 8	2 (−5.8 to 1.6)	0.268
Cross-clamp time, minutes	78 ± 23	81 ± 24	3 (−14 to 8)	0.598
Cardiopulmonary bypass time, minutes	98 ± 25	102 ± 25	4 (−15 to 8)	0.540
Procedure time, minutes	235 ± 45	235 ± 41	0 (−20 to 20)	0.997

Abbreviations: SD, standard deviation.

**Table 2:** Analgesic consumption and pain scores.

	Control	Case	Mean Difference	*p*-Value
	Mean ± SD	Mean ± SD	(95% CI)	
Total OME, mg	461 ± 185	224 ± 108	237 (172 to 304)	<0.001
Total acetaminophen, mg	9947 ± 3086	9370 ± 4861	577 (−1480 to 2633)	0.574
Intraoperative ketamine used	3 (6%)	0 (0%)	-	0.479
Postoperative OME, mg	238 ± 154	190 ± 104	48 (−10 to 107)	0.104
Postoperative gabapentin, mg	1708 ± 827	1375 ± 480	333 (38 to 628)	0.027
Postoperative NSAIDS used	8 (16%)	3 (10.7%)	-	0.761
	**Control**	**Case**	**Median Difference**	***p*-Value**
	**Median (IQR)**	**Median (IQR)**	**(95% CI)**	
Intraoperative OME, mg	223.6 (188–259)	34.1 (27.5–40.7)	−165 (−170 to −155)	<0.001
Highest reported pain score, POD #0	8 (7.0–9.0)	8 (7.0–9.5)	3.6 × 10^−5^ (−1.9 × 10^−5^ to 1.0)	0.246
Highest reported pain score, POD #1	8 (7.0–9.75)	8 (7.0–9.25)	−6.6 × 10^−5^ (−1.0 to 1.0)	0.949
Highest reported pain score, POD #2	7 (5.25–8.0)	6 (5.0–8.0)	−4.6 × 10^−5^ (−1.0 to 1.0)	0.442
Highest reported pain score, POD #3	6 (3.25–7.0)	4.5 (3.0–6.0)	−1.0 (−2.0 to 1.6 × 10^−5^)	0.139
Highest reported pain score, POD #4	5 (0.0–7.0)	4 (0.0–6.0)	−2.6 × 10^−5^ (−2.0 to 8.9 × 10^−6^)	0.423
Highest reported pain score, POD #5	4 (0.0–6.0)	5 (0.0–6.25)	7.0 × 10^−5^ (−1.0 to 3.0)	0.637

Abbreviations: SD, standard deviation; CI, confidence interval; OME, oral morphine equivalent IQR, interquartile range; POD, postoperative day.

## Data Availability

Data available upon request this section.
